# Relationship between 25-hydroxy vitamin D and knee osteoarthritis: a systematic review and meta-analysis of randomized controlled trials

**DOI:** 10.3389/fmed.2023.1200592

**Published:** 2023-08-02

**Authors:** Rui Wang, Zheng-ming Wang, Si-cheng Xiang, Zhao-kai Jin, Jing-jing Zhang, Ji-cheng Zeng, Pei-jian Tong, Shuai-jie Lv

**Affiliations:** ^1^Guanghua Clinical Medical College, Shanghai University of Traditional Chinese Medicine, Shanghai, China; ^2^Department of Orthopedic Surgery, Shanghai Guanghua Hospital of Integrated Traditional Chinese and Western Medicine, Shanghai, China; ^3^Shi's Center of Orthopedics and Traumatology, Shuguang Hospital Affiliated to Shanghai University of Traditional Chinese Medicine, Shanghai, China; ^4^The First Clinical College, Zhejiang Chinese Medical University, Hangzhou, Zhejiang, China; ^5^The First Affiliated Hospital of Zhejiang Chinese Medical University (Zhejiang Provincial Hospital of Chinese Medicine), Hangzhou, Zhejiang, China

**Keywords:** 25-hydroxyvitamin D, vitamin D, randomized controlled trial, meta-analysis, knee osteoarthritis

## Abstract

**Objective:**

In order to examine the relationship between 25-hydroxyl vitamin D and knee osteoarthritis (KOA), a meta-analysis of 8 randomized controlled trials (RCTs) publications was hereby performed.

**Methods:**

For the purpose of finding pertinent research, the databases of PubMed, Embase and the Cochrane Library were searched. Factors including tibial cartilage volume, joint space width (JSW), synovial fluid volume, and Western Ontario and McMaster Universities Arthritis Index (WOMAC) were correspondingly evaluated, and the results were expressed using SMD and 95% confidence intervals (CI).

**Results:**

The present meta-analysis evaluated the effects of vitamin D supplementation in patients with knee osteoarthritis, with 3,077 patients included. The results showed that vitamin D administration had a statistically significant impact on the amount of synovial fluid, Visual Analog Scale (VAS) and tibial cartilage. The pain and function scales of the WOMAC scale presented a statistically significant difference, and there was no discernible difference between the vitamin D and placebo groups in the stiffness scale. Additionally, bone marrow lesions and alterations in the diameter of the joint space were not influenced by the administration of vitamin D, and according to a subgroup study, a daily vitamin D supplement containing more than 2,000 IU significantly slowed the development of synovial tissue.

**Conclusion:**

Vitamin D supplementation did benefit those suffering from knee discomfort and knee dysfunction.

**Systematic review registration:**

https://www.crd.york.ac.uk/prospero/display_record.php?ID=CRD42022332033, identifier: CRD42022332033.

## Graphical Abstract

This image presents the methods, interventions and main results of this study. 
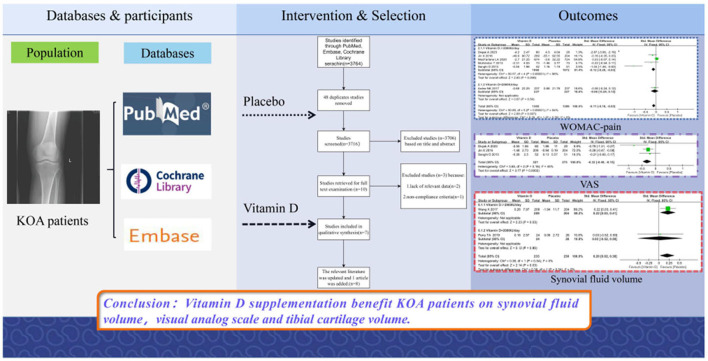

## Introduction

An estimated 14 million Americans have been reported to suffer from symptomatic knee osteoarthritis (KOA), a chronic degenerative joint condition. KOA is the most prevalent degenerative joint disease and is accompanied by pain, dysfunction, joint space constriction and cartilage deterioration. It is primarily defined by cartilage degradation, loss of joint space, osteophyte growth and subchondral bone changes (1). The prevalence of knee osteoarthritis is as high as 40% among 70–74-year-olds (2), and age, sex, heredity as well as lifestyle factors (alcohol, tobacco) are closely related to osteoarthritis (3). Besides, surgery is usually considered the best choice for patients with advanced KOA, while oral medications, nutritional supplementation, dry needling (4), therapeutic exercise, manual therapy (5) and pain education (6) are also good options for those with early to mid-stage KOA.

Generally, vitamin D comes in two forms, i.e., ergocalciferol (vitamin D^2^) and cholecalciferol (vitamin D^3^), both of which are converted to 25-hydroxyvitamin D in the liver. The kidneys transform 25-hydroxyvitamin D to 1,25-dihydroxyvitamin D, the steroid hormones that mediate the biological actions of vitamin D (7).

The present findings revealed that 1,25-dihydroxyvitamin D reduced cartilage degradation and osteophyte development in the osteoarthritis mice by decreasing the release of inflammatory cytokines (8).

There may be a relationship between vitamin D and pain (9). Studies have demonstrated the association of vitamin D deficiency with KOA progression, but these findings are not satisfactory in terms of support for trace element replacement. In clinical studies, vitamin D supplementation has been shown to improve the life quality and physical performance in patients with osteoarthritis by enhancing the pain relief effects of vitamin D and KOA (10). However, knee pain or cartilage volume loss in sufferers with symptomatic KOA was not decreased (11). Herein, it was suggested that the effect of vitamin D on knee osteoarthritis was still unclear, so an analysis of articles from published randomized controlled trials (RCTs) was carried out to assess its effects.

## Methods

### Data and sources and search strategy

The meta-analysis protocol was prospectively registered on PROSPERO (CRD42022332033), and this meta-analysis were conducted in accordance with the preferred reporting items for systematic reviews and meta-analyses (PRISMA) guidelines.

Data from PubMed, Embase, and Cochrane Library up to 23 December 2021 were searched for randomized controlled trials with no language restrictions. By June 25, 2023, one new relevant literature had been updated and included. The combination of subject words with free word retrieval, which could be found in the Appendix, was used as a search strategy.

### Study selection criteria

Studies were included in accordance with Populations, Interventions, Comparison, Outcomes and Study Design (PICOS).

#### Population

Patients diagnosed with KOA underwent supplementation with vitamin D. Reasons for exclusion included: secondary OA, inflammatory arthritis, osteoporotic fracture, previous knee surgery or arthroscopy within 6 months, rheumatoid or psoriatic arthritis, lupus or cancer.

#### Intervention and comparison

Patients in the treatment group were given a vitamin D supplementation. There are no requirements for the type of vitamin D preparation to be taken, the dose or the duration of treatment. Patients in the control group received an identical inert placebo.

#### Outcomes

The primary outcomes included the Western Ontario and McMaster Universities Arthritis Index (WOMAC), Visual Analog Scale (VAS), changes in tibial cartilage volume, etc., while the secondary outcomes included general information about the follow-up, the level of 25-hydroxyvitamin D, etc.

#### Study design

RCTs were included if sufficient outcomes data for both placebo and vitamin D group were reported in the study. All conference articles, mini review, cases reports, letters, reviews, case control studies and cohort studies were excluded. Non-English studies were excluded as well.

### Data extraction

Data were hereby extracted using a pre-generated data table, with items including general information, year of publication, country, follow-up time, main outcomes, etc. included. Eligible studies were selected by two researchers who independently screened the study titles and abstracts. Disagreements were discussed and resolved by a third researcher to decide whether to include or exclude a study. Finally, three researchers extracted the data from eligible studies into a data entry table.

### Risk of bias assessment

Upon the completion of data extraction, the quality of each study undergoing was assessed using the Cochrane risk of bias tool, and disagreements were resolved by consensus or appeal to a third researcher. Random allocation sequence, allocation scheme concealment, blinding procedure, selective reporting, and other bias were also assessed. Each article was rated as having a high, low or unclear risk of bias.

### Sensitivity analysis and publication bias

Considering a small heterogeneity among studies, sensitivity analysis was not carried out, and publication bias testing was not performed due to an insufficient number of included studies.

### Statistical analysis

The meta-analysis was performed using Review Manager 5.3.5 software (The Cochrane Collaboration, Oxford, UK). Standardized mean difference (SMD) and 95% confidence interval (CI) were applied for effect magnitude representing the same outcome for different units. The Cochran Q statistic (significance level of *P* < 0.10) and *I*^2^ statistics (significance level of 50%) were used to assess the heterogeneity (12). Low, medium and high heterogeneity were found for the I^2^ values of 25, 50, and 75%, respectively (13). Based on daily supplemental vitamin doses, subgroup analyses were further carried out.

## Results

### Search results

**Figure 1:**
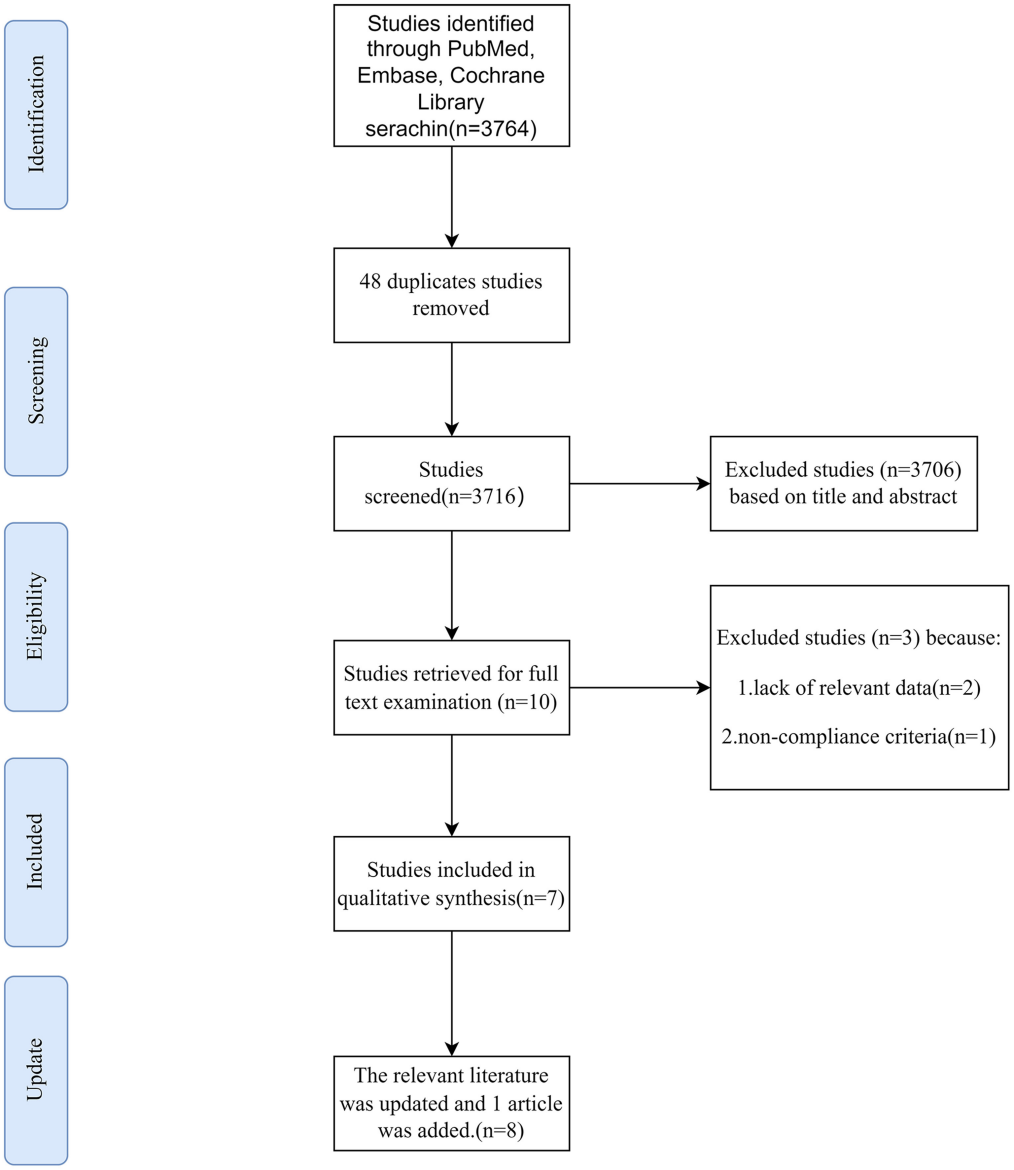
Selection of studies for inclusion in the meta-analysis.

The study selection process is shown in Figure 1. A total of 3,764 articles were obtained according to the retrieval strategy, among which, 48 duplicated were deleted and 3,716 were included in the screening. Subsequently, 3,706 articles were discarded on the basis of titles and abstracts, and 10 were accepted for further screening. Three publications were removed after reading the full text due to lack of relevant data to extract and inconsistent standards. As of 23 December 2021, 7 articles were finally included. Currently, one more relevant literature has been updated and added.

### Research characteristic and quality assessment

The baseline features of the selected studies are shown in Table 1. A total of 8 articles published between 2013 and 2023 were included in the meta-analysis, with 3,077 patients enrolled, among which, 1,538 received vitamin D therapy and 1,539 received placebo. Herein, 36.5% of the patients were males, and 63.5% were females. The mean age of the vitamin D group was 61.8 years, while that of the placebo group was 61.7 years. The average BMI of vitamin D was 29.5, while that of the placebo group was 29.4.

**Table 1:** Summary characteristics of studies and participants.

**Studies**	**Study design**	**Country**	**Follow-up (month)**	**Primary outcomes**	**Treatment regimen**	**No.of patient**	**Gender (male/female)**	**Age (year)**	**BMI (kg/m2)**
McAlindon T	RCT	USA	24	WOMAC pain, and cartilage volume loss	≥2,000 IU/day Vitamin D^a^	73	24/49	61.8 ± 7.7	30.5 ± 5.0
					Placebo	73	33/40	63.0 ± 9.3	30.8 ± 6.4
Ardne NK	RCT	England	36	Radiological progression	800 IU/day Vitamin D	237	93/144	64.0 ± 8.0	30.0 ± 5.0
					Placebo	237	92/145	64.0 ± 8.0	29.0 ± 5.0
Jin XZ	RCT	Australia	24	WOMAC score, VAS, and tibial cartilage volume	50,000 IU monthly Vitamin D3	209	103/106	63.5 ± 6.9	29.6 ± 5.4
					Placebo	204	102/102	62.9 ± 7.2	29.6 ± 4.6
Sanghi	RCT	India	12	Knee pain and function	≥2,000 IU/day Vitamin D^b^	52	16/36	53.2 ± 9.6	25.9 ± 2.5
					Placebo	51	21/30	53.0 ± 7.4	25.7 ± 2.6
Wang X	RCT	Australia	24	Cartilage volume changes	50,000 IU monthly Vitamin D3	209	51/158	63.6 ± 6.9	29.6 ± 5.4
					Placebo	204	50/154	62.9 ± 7.2	29.6 ± 4.6
Perry TA	RCT	England	36	Rate of joint space narrowing and WOMAC score	800 IU/day Vitamin D	24	7/17	63.0 ± 5.8	28.2 ± 4.0
					Placebo	26	6/20	63.6 ± 7.2	29.2 ± 5.6
MacFarlane LA	RCT	USA	60	WOMAC score	2,000 IU/day vitamin D3	674	235/439	67.5 ± 6.7	31.8 ± 7.0
					Placebo	724	243/481	68.0 ± 7.1	31.8 ± 7.9
Divjak A	RCT	Serbia	3	WOMAC score, VAS	4,000 IU/day vitamin D3	80	47/33	57.1 ± 4.3	30.2 ± 2.8
					Placebo				

a 2,000 IU/day vitamin D with subsequent adjustment in 2000 IU increments at the 4, 8, and 12 months.

b 60,000 IU/day vitamin D for 10 days, followed by 60000 IU monthly vitamin D.

RCT, randomized controlled trial; WOMAC, Western Ontario and McMaster Universities Arthritis Index; VAS, visual analog scale.

**Figure 2:**
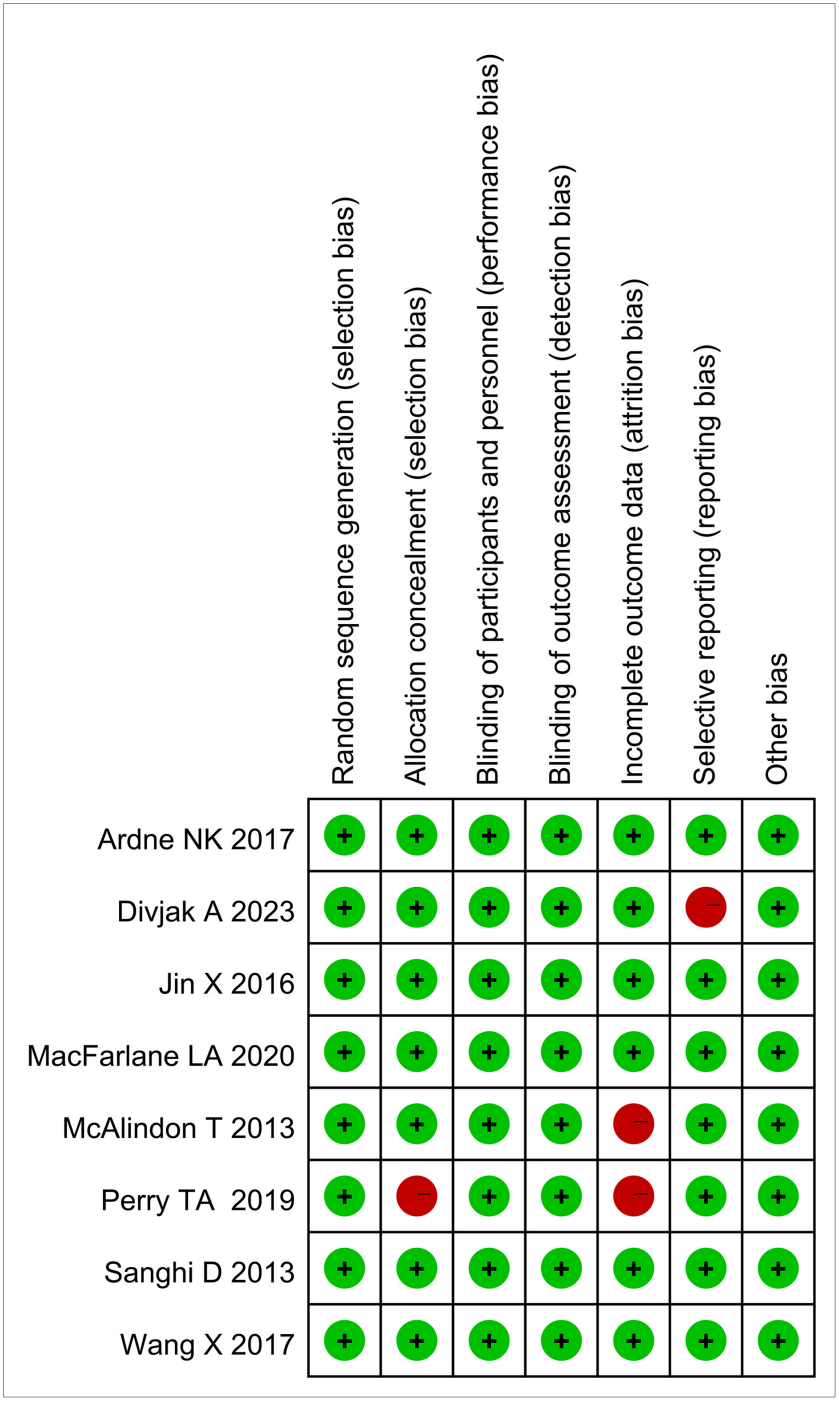
Risk of bias summary.

These articles were randomized controlled studies, which were conducted in the United Kingdom, India, Serbia, Australia and the United States. The length of follow-up ranged from 3 to 60 months, and none of these RCTs were found to be at high risk of bias. Figure 2 shows the results of the quality assessment included in this article.

### Changes in tibial cartilage volume

**Figure 3:**
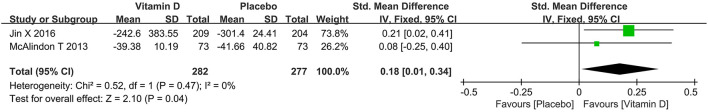
The forest plot for studies on the effect of vitamin D on the tibial cartilage volume.

The Jin X and Mcalidon T studies demonstrated the correlation between vitamin D and tibial cartilage volume. The present forest plot (Figure 3) presented a statistical significance (SMD = 0.18, 95% CI: 0.01 to 0.34, *P* = 0.04) in the tibial cartilage volume between the two studies, and the heterogeneity between two studies was small (*I*^2^ = 0%).

### Changes in VAS

**Figure 4:**
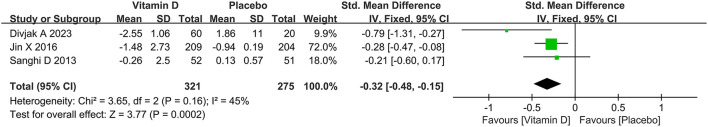
The forest plot for studies on the effect of vitamin D on the VAS. VAS, visual analog scale.

A decrease in VAS was observed in both the vitamin D and placebo groups. As shown in Figure 4, vitamin D supplementation was beneficial for pain relief in patients with KOA (SMD = −0.32, 95% CI: −0.48 to −0.15, *P* = 0.0002), presenting a mild heterogeneity (*I*^2^ = 45%).

### Change in synovial fluid volume

**Figure 5:**
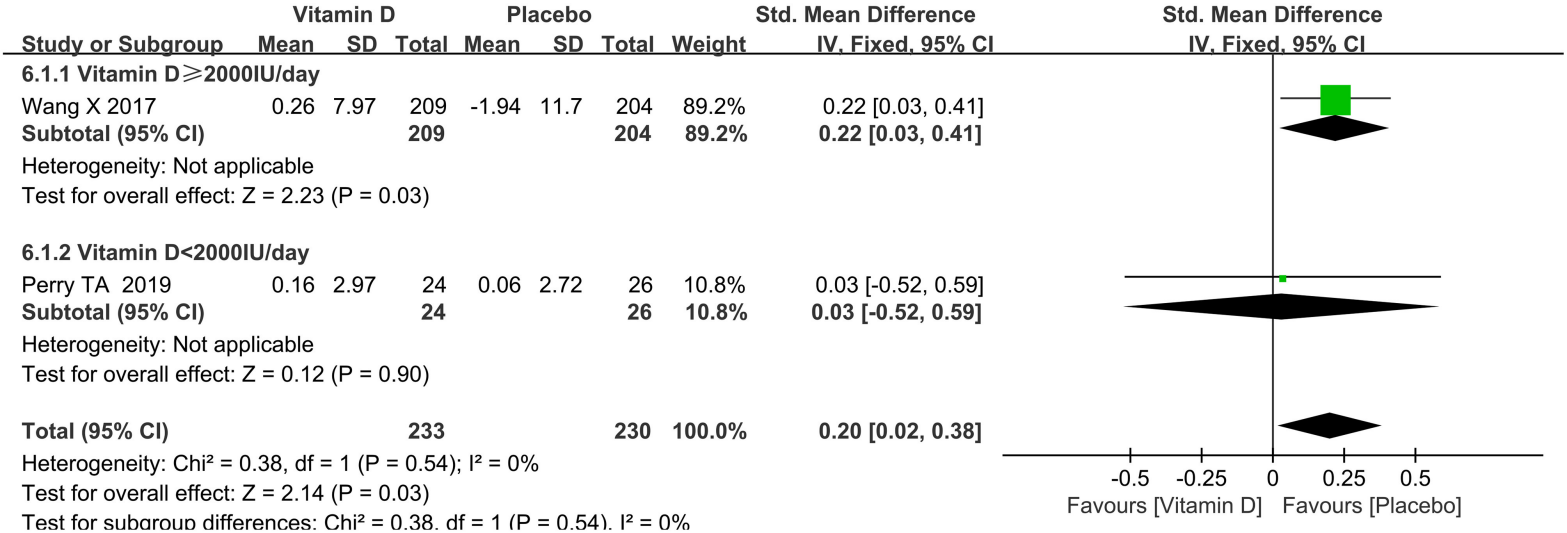
The forest plot for studies on the effect of vitamin D on the synovial fluid volume.

It could be observed from Figure 5 that the synovial fluid volume in the placebo group was larger than that in the vitamin D group (SMD = 0.20, 95% CI: 0.02 to 0.38, *P* = 0.03), and that there was no heterogeneity (*I*^2^ = 0%).

### Changes in WOMAC scores

**Figure 6:**
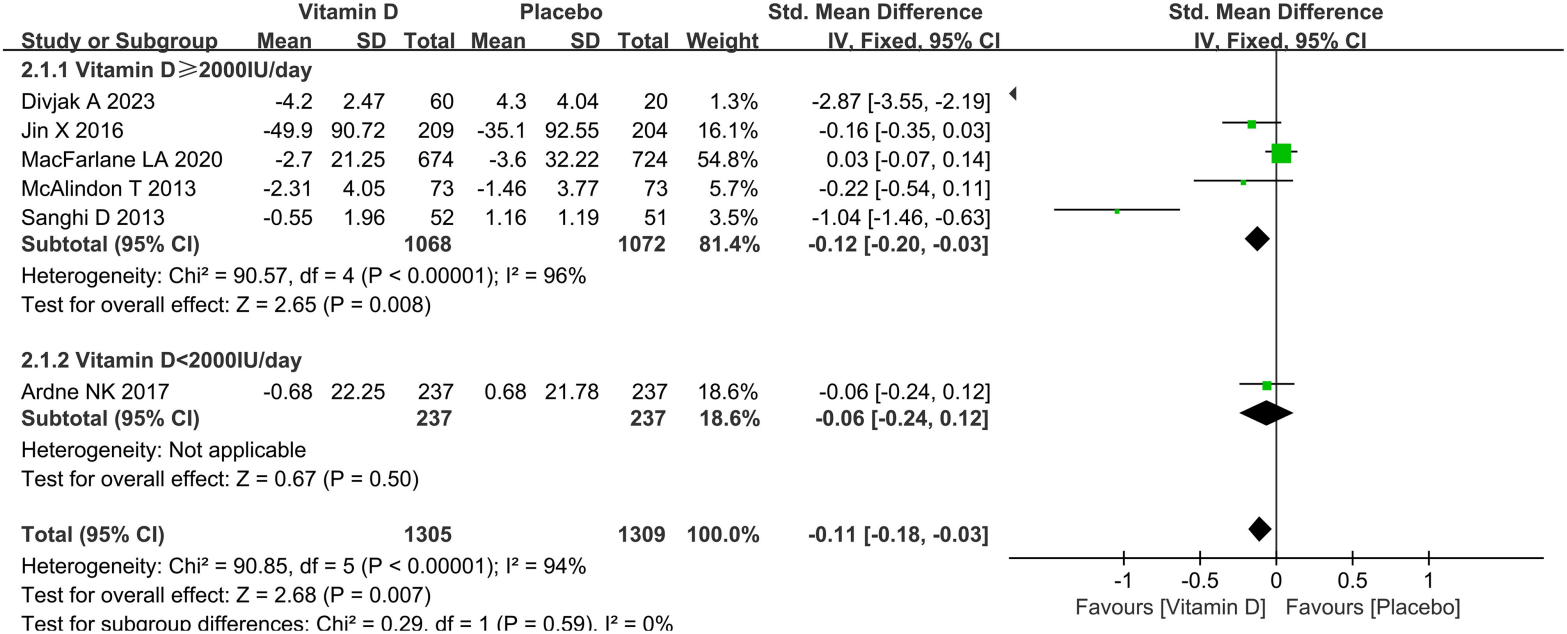
The forest plot for studies on the effect of vitamin D on the change of WOMAC pain. WOMAC, Western Ontario and McMaster Universities Arthritis Index.

**Figure 7:**
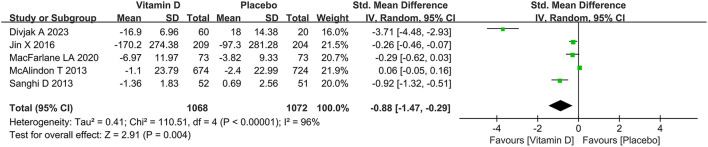
The forest plot for studies on the effect of vitamin D on the change of WOMAC function. WOMAC, Western Ontario and McMaster Universities Arthritis Index.

**Figure 8:**
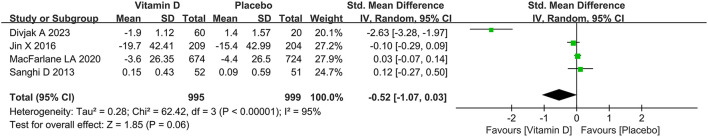
The forest plot for studies on the effect of vitamin D on the change of WOMAC stiffness. WOMAC, Western Ontario and McMaster Universities Arthritis Index.

Herein, six studies were analyzed for WOMAC pain, five for WOMAC function and four for WOMAC stiffness, with the results shown in Figures 6–8, respectively. As shown in Figure 6, vitamin D supplementation had a statistical significance on WOMAC pain relief (SMD = −0.11, 95% CI: −0.18 to −0.03, *P* = 0.007), and the heterogeneity was relatively small (*I*^2^ = 0%). Meanwhile, it could be seen from Figure 7 that vitamin D did not have a statistically significant effect on the WOMAC stiffness score (SMD = −0.52, 95% CI: −1.07 to 0.03, *P* = 0.06), and the results among studies were heterogeneous (*I*^2^ = 95%). Additionally, Figure 8 demonstrated a statistically significant difference between the vitamin D and placebo groups in WOMAC function (SMD = −0.88, 95% CI: −1.47 to −0.29, *P* = 0.004), and there was obvious heterogeneity (*I*^2^ = 96%).

### Change in joint space width

**Figure 9:**
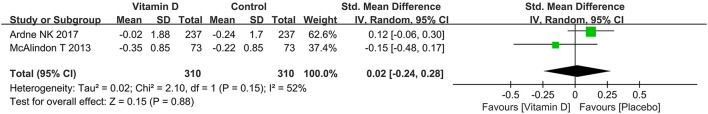
The forest plot for studies on the effect of vitamin D on the change of joint space width.

There was no significant difference between the two groups in changes in joint space width when the results were pooled (SMD = 0.02, 95% CI: −0.24 to 0.28, *P* = 0.15), and the heterogeneity test showed moderate heterogeneity (*I*^2^ = 52%), as shown in Figure 9.

### Change in bone marrow lesions

**Figure 10:**
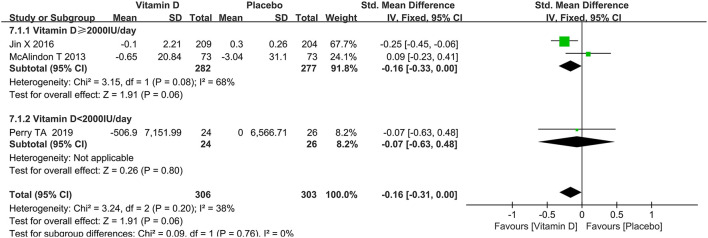
The forest plot for studies on the effect of vitamin D on the change of bone marrow lesions.

Data on bone marrow lesions were reported in three studies. As shown in Figure 10, there was no significant statistical significance in changes in bone marrow lesions between the two groups (SMD = −0.16, 95% CI: −0.31 to 0.00, *P* = 0.06), and no significant heterogeneity was observed (*I*^2^ = 38%).

### Subgroup analysis

Subgroup analyses of WOMAC pain, synovial fluid volume and bone marrow lesions were conducted based on different dosages of vitamin D intake. A subgroup analysis of the dosage of vitamin D revealed that a higher vitamin D contributed to slowing down the progression of the synovial fluid volume. The results of the subgroup analysis showed that the group taking higher doses of vitamin D played a greater role in reducing WOMAC pain scores. Besides, as presented in Table 2, there was no statistically significant difference in the effect of dose on bone marrow lesions.

**Table 2:** Subgroup analysis of the relationship between vitamin D dosage and some clinical outcomes.

**Items**	**Subgroup**	**Heterogeneity**		**Meta-analysis**		**Number of studies**	***I^2^* of test for subgroup difference**
		***I** ^2^*	***P*** **-value**	**SMD**	**95% CI**		
Bone marrow lesions	Vitamin D ≥ 2,000 IU/day	68.0%	0.08	−0.16	−0.33 to 0.00	2	0.0%
	Vitamin D < 2,000 IU/day	NA	NA	−0.07	−0.63 to 0.48	1	
Synovial fluid volume	Vitamin D ≥ 2,000 IU/day	NA	NA	0.22	0.03 to 0.41	1	0.0%
	Vitamin D < 2,000 IU/day	NA	NA	0.03	−0.52 to 0.59	1	
WOMAC pain	Vitamin D ≥ 2,000 IU/day	96.0%	< 0.00001	−0.12	−0.20 to −0.03	5	0.0%
	Vitamin D < 2,000 IU/day	NA	NA	−0.06	−0.24 to 0.12	1	

WOMAC, Western Ontario and McMaster Universities Arthritis Index; SMD, standardized mean difference; CI, confidence interval; NA, not applicable.

### Sensitivity analysis and publication bias

Due to a small heterogeneity among studies, sensitivity analysis on the tibial cartilage volume, VAS, and synovial fluid volume was not carried out, and there was no statistical test for publication bias because of the limited number of studies.

## Discussions

Herein, the meta-analysis evaluated how vitamin D supplementation affected KOA patients, and found that vitamin D administration might benefit KOA patients by reducing the increase of the synovial fluid volume, improving articular pain and function and increasing the tibial cartilage volume. Besides, supplementing with vitamin D showed no positive impact on bone marrow lesions, joint space width or joint stiffness.

Some previous research has shown the effectiveness of vitamin D supplementation in reducing pain and improving the function in patients with knee osteoarthritis (9, 14, 15). A meta-analysis reported that vitamin D administration was helpful in the improvement of WOMAC pain and function in patients with KOA (16). However, some randomized controlled trials showed the opposite. In a large sample of US adults, a study by MacFarlane et al. found that vitamin D supplementation for an average of 5.3 years did not reduce knee pain, improve function, or reduce stiffness (17).

The results of the present meta-analysis showed that vitamin D could affect the VAS of patients. Sanghi (18) and colleagues demonstrated that vitamin D supplementation was associated with reduced knee pain at 12 months, but the association was unlikely to be clinically relevant. It was hereby believed that although the VAS of the patients had improved, the reliability of this result should still be further confirmed in subsequent studies. The WOMAC pain scale was more specific and more useful for patients to score their pain levels. The statistical analysis showed that vitamin D supplementation did contribute to the relief of WOMAC pain and the functional improvement in patients with KOA. The improvement in joint function and WOMAC pain reinforced the ability of the vitamin to relieve the pain and improve the life function in patients with KOA.

The current data were consistent with previous studies failing to provide significant improvement in bone marrow lesion (BML) and joint space width. Data from Perry et al. showed that supplementing with 800 IU of vitamin D per day for 2 years resulted in no appreciable reduction in subchondral BML in individuals with symptomatic KOA over these 2 years.

Data from McAlindon et al. (19) suggested that vitamin D therapy had no impact on structural modifications in males or females with symptomatic KOA. Besides, a meta-analysis by Zhao ZX found that there was no statistical evidence of a difference in the change in joint space width between the treatment and control groups (20), which was also supported by the present pooled results.

In comparison to those of Perry et al., the present results showed that vitamin D could slow down the increase of the synovial tissue volume (21). According to the research, vitamin D could delay the degeneration of tibial cartilage volume, which is consistent with the results of Malas FU (22) and Ding C (23), but contrary to those of the meta-analysis of Yu Y (24).

Stimulation of Toll-Like Receptor 2 (TLR-2), which enhanced the release of prostaglandin E2, Matrix Metalloproteinases (MMPs) and nitric oxide, was demonstrated by vitamin D deficiency during the progression of osteoarthritis. Likewise, the upregulation of Tumor Necrosis Factor-α, Interleukin-1β and MMP-3 gene expression was driven by TLR-4 (25, 26). Vitamin D could reduce inflammation, fatty infiltration and cartilage loss in the knee of hyperlipidemic microswine (27). Besides, low vitamin D caused quadriceps dysfunction, and the vitamin D deficiency was closely related with quadriceps strength, demonstrating vitamin D deficiency as a cause of aggravation of knee pain (28). The above mechanisms may explain the results found in the current meta-analysis.

### Future directions and clinical implications

This research showed that vitamin D supplements might be beneficial for patients presenting knee osteoarthritis pain symptoms, but might not contribute to radiographic changes. In the future, in-depth research on the serum vitamin D content as well as different vitamin D preparations, treatment courses and dosages should be conducted.

### Advantages and disadvantages

Randomized controlled studies were included in this meta-analysis, and the quality of the evidence was relatively high. However, there are still several limitations to this meta-analysis. Firstly, due to the relatively small number of articles included in the meta-analysis, there was a lack of publication bias and sensitivity analysis. Secondly, the lack of original data in some studies and different related index units may affect the pooled results.

## Conclusions

A meta-analysis involving 8 RCTs supported that vitamin D was beneficial in slowing down the progression of the synovial fluid volume, improving the subjective pain and function of the patients and reducing the tibial cartilage degeneration. However, more high-quality studies are needed to verify the hereby-obtained results.

## Data availability statement

The original contributions presented in the study are included in the article/Supplementary material, further inquiries can be directed to the corresponding author.

## Author contributions

RW and Z-mW drafted the manuscript. S-cX collected and analyzed the data. S-jL and P-jT made the study design. J-jZ, J-cZ, and S-jL revised and supervised the manuscript. All authors contributed to the article and approved the submitted version.
