# Supplementary material for: Relationship between 25-hydroxy vitamin D and knee osteoarthritis: a systematic review and meta-analysis of randomized controlled trials
Source: Front Med (Lausanne). 2023 Aug 2;10:1200592. doi: 10.3389/fmed.2023.1200592 (PMC10433223; doi:10.3389/fmed.2023.1200592)
Supplement: Supplementary file 2 [file Table_2.DOC]

Pubmed serach strategy

(("osteoarthritis, knee"[MeSH Terms] OR ("osteoarthritis, knee"[MeSH Terms] OR ("osteoarthritis"[All Fields] AND "knee"[All Fields]) OR "knee osteoarthritis"[All Fields] OR ("knee"[All Fields] AND "osteoarthritides"[All Fields])) OR ("osteoarthritis, knee"[MeSH Terms] OR ("osteoarthritis"[All Fields] AND "knee"[All Fields]) OR "knee osteoarthritis"[All Fields] OR ("knee"[All Fields] AND "osteoarthritis"[All Fields])) OR ("osteoarthritis, knee"[MeSH Terms] OR ("osteoarthritis"[All Fields] AND "knee"[All Fields]) OR "knee osteoarthritis"[All Fields] OR ("osteoarthritis"[All Fields] AND "knee"[All Fields]) OR "osteoarthritis of knee"[All Fields]) OR ("osteoarthritis, knee"[MeSH Terms] OR ("osteoarthritis"[All Fields] AND "knee"[All Fields]) OR "knee osteoarthritis"[All Fields] OR ("osteoarthritis"[All Fields] AND "knee"[All Fields]) OR "osteoarthritis of the knee"[All Fields])) AND ("Calcifediol"[MeSH Terms] OR ("Calcifediol"[MeSH Terms] OR "Calcifediol"[All Fields] OR "25 hydroxyvitamin d 3"[All Fields]) OR ("Calcifediol"[MeSH Terms] OR "Calcifediol"[All Fields] OR "25 hydroxyvitamin d 3"[All Fields]) OR ("Calcifediol"[MeSH Terms] OR "Calcifediol"[All Fields]) OR ("Calcifediol"[MeSH Terms] OR "Calcifediol"[All Fields]) OR ("Calcifediol"[MeSH Terms] OR "Calcifediol"[All Fields]) OR ("Calcifediol"[MeSH Terms] OR "Calcifediol"[All Fields] OR "25 hydroxyvitamin d3"[All Fields]) OR ("Calcifediol"[MeSH Terms] OR "Calcifediol"[All Fields] OR "25 hydroxyvitamin d3"[All Fields]) OR ("Calcifediol"[MeSH Terms] OR "Calcifediol"[All Fields] OR "calcidiol"[All Fields]) OR ("Calcifediol"[MeSH Terms] OR "Calcifediol"[All Fields] OR "25 hydroxycholecalciferol"[All Fields]) OR ("Calcifediol"[MeSH Terms] OR "Calcifediol"[All Fields] OR "25 hydroxycholecalciferol"[All Fields]) OR ((("Calcifediol"[MeSH Terms] OR "Calcifediol"[All Fields]) AND ("3"[All Fields] AND ("beta"[Journal] OR "beta"[All Fields]) AND "5E"[All Fields] AND "7E"[All Fields])) AND ("isomerism"[MeSH Terms] OR "isomerism"[All Fields] OR "isomer"[All Fields] OR "isomers"[All Fields])) OR ("Calcifediol"[MeSH Terms] OR "Calcifediol"[All Fields] OR ("Calcifediol"[All Fields] AND "anhydrous"[All Fields])) OR ("Calcifediol"[MeSH Terms] OR "Calcifediol"[All Fields] OR ("anhydrous"[All Fields] AND "Calcifediol"[All Fields])) OR ("Calcifediol"[MeSH Terms] OR "Calcifediol"[All Fields] OR "dedrogyl"[All Fields]) OR ("Calcifediol"[MeSH Terms] OR "Calcifediol"[All Fields] OR "hidroferol"[All Fields]) OR ((("Calcifediol"[MeSH Terms] OR "Calcifediol"[All Fields]) AND ("3"[All Fields] AND ("alpha"[All Fields] OR "alpha s"[All Fields] OR "alphas"[All Fields]) AND "5Z"[All Fields] AND "7E"[All Fields])) AND ("isomerism"[MeSH Terms] OR "isomerism"[All Fields] OR "isomer"[All Fields] OR "isomers"[All Fields])) OR ("Calcifediol"[MeSH Terms] OR "Calcifediol"[All Fields] OR "calderol"[All Fields]) OR ("25-Hydroxyvitamin D 2"[MeSH Terms] OR ("25-Hydroxyvitamin D 2"[MeSH Terms] OR "25-Hydroxyvitamin D 2"[All Fields] OR "25 hydroxycalciferol"[All Fields]) OR ("25-Hydroxyvitamin D 2"[MeSH Terms] OR "25-Hydroxyvitamin D 2"[All Fields] OR "25 hydroxycalciferol"[All Fields]) OR ("25-Hydroxyvitamin D 2"[MeSH Terms] OR "25-Hydroxyvitamin D 2"[All Fields] OR "ercalcidiol"[All Fields]) OR ("25-Hydroxyvitamin D 2"[MeSH Terms] OR "25-Hydroxyvitamin D 2"[All Fields] OR "25 hydroxyvitamin d2"[All Fields]) OR ("25-Hydroxyvitamin D 2"[MeSH Terms] OR "25-Hydroxyvitamin D 2"[All Fields] OR "25 hydroxyvitamin d2"[All Fields]) OR ("25 hydroxyvitamin d"[Supplementary Concept] OR "25 hydroxyvitamin d"[All Fields] OR "25 hydroxyergocalciferol"[All Fields] OR "25-Hydroxyvitamin D 2"[MeSH Terms] OR "25-Hydroxyvitamin D 2"[All Fields]) OR ("25 hydroxyvitamin d"[Supplementary Concept] OR "25 hydroxyvitamin d"[All Fields] OR "25 hydroxyergocalciferol"[All Fields] OR "25-Hydroxyvitamin D 2"[MeSH Terms] OR "25-Hydroxyvitamin D 2"[All Fields]) OR ("25-Hydroxyvitamin D 2"[MeSH Terms] OR "25-Hydroxyvitamin D 2"[All Fields] OR "25-Hydroxyvitamin D 2"[All Fields])))) AND (clinicaltrial[Filter])

Embase

No. Query Results

#5. #1 AND #4

#4. #2 OR #3

#3. '25 hydroxyergocalciferol'/exp

#2. 'calcifediol blood level'/exp OR

'calcifediol'/exp

#1. 'knee osteoarthritis'/exp

Cochrane Library

#1 MeSH descriptor: [Osteoarthritis, Knee] explode all trees

#2 Knee Osteoarthritides OR Knee Osteoarthritis OR Osteoarthritis of Knee OR Osteoarthritis of the Knee

#3 MeSH descriptor: [Osteoarthritis, Knee] explode all trees

#4 (25 Hydroxyvitamin D 3) OR (25 Hydroxycholecalciferol Monohydrate)OR(25 Hydroxyvitamin D3) OR (Calcidiol) OR (25 Hydroxycholecalciferol) OR (Calcifediol Anhydrous)OR(Anhydrous,Calcifediol)OR(Dedrogyl)OR(Hidroferol)OR(Calderol)

#5 MeSH descriptor: [25-Hydroxyvitamin D 2] explode all trees #6 25 Hydroxycalciferol OR Ercalcidiol OR 25 Hydroxyvitamin D2 OR 25 Hydroxyergocalciferol OR 25 Hydroxyvitamin D 2

#7 #1 OR #2

#8 #3 OR #4

#9 #5 OR #6

#10 #8 OR #9

#11 #7 AND #10

#12 randomized controlled trial

#13 #11 AND #12
